# Melasma: A Condition of Asian Skin

**DOI:** 10.7759/cureus.14398

**Published:** 2021-04-10

**Authors:** Michelle X Wu, Ruth Antony, Harvey N Mayrovitz

**Affiliations:** 1 Osteopathic Medicine, Nova Southeastern University Dr. Kiran C. Patel College of Osteopathic Medicine, Davie, USA; 2 Medical Education, Nova Southeastern University Dr. Kiran C. Patel College of Allopathic Medicine, Davie, USA

**Keywords:** melasma, asian skin conditions, pathophysiology, risk factors, treatments, prevention, complications

## Abstract

Melasma is a benign yet psychologically debilitating skin condition that is prevalent among women of darker phenotypes, and particularly in Asian women. This potentially preventable condition can lead to skin discoloration that is hard to treat and can frequently recur. This review aims to (1) highlight the pathophysiology of melasma, (2) describe its important risk factors, and (3) identify prevention methods, available treatment options, and potential complications of melasma. By raising awareness of this condition, we hope that physicians and patients will be able to be better informed to discuss screening options and to avoid preventable risk factors, especially in patients who are predisposed to the disease.

## Introduction and background

Health disparity is very prevalent in dermatology. When examining skin conditions, many disorders are missed particularly among Asians, Hispanics, and Blacks due to their darker, ethnic skin. Asian Americans in particular, represent the fastest-growing minority group in the United States but are under-represented in dermatologic patient populations [1]. The most common of these missed diagnoses include acne and eczema, followed by other pigmentation disorders including melasma [2]. Melasma is an acquired hyperpigmentation skin disorder that typically affects sun-exposed areas of the skin, mainly facial areas with women of Fitzpatrick skin phototype III-VI [3,4]. The clinical presentation of melasma is generally an asymptomatic light to dark brown hyperpigmentation with irregular borders [5]. Despite the border irregularity, it is considered a benign process. Asian populations, in particular, consider pigmentation abnormalities such as melasma more important in the aging process, rather than wrinkles [6]. Rosacea is a benign condition, similar to melasma, seen in patients of lighter skin tones [7]. However, it has been more extensively characterized in comparison to melasma. As the epidemiology and pathophysiology of melasma have not been intensively studied, it is important to characterize this prevalent pigmentary disorder. The purpose of this article is to document and clarify the etiology, pathophysiology, treatment, outcomes, and complications of melasma with an emphasis on Asian skin. Our goal is to bring together evidence on the current understanding of melasma to aid in decreasing the number of missed clinical diagnoses.

Preliminary results were previously presented at the 2021 Florida Osteopathic Medical Association Annual Research Poster Competition. 

## Review

Multiple databases have been searched in conjunction with this review. All authors have critically assessed these as to suitability for inclusion.

Methods

English language literature searches for years 2010-2020 were done via PubMed, CINAHL Complete, and Biomedical Reference Collection: Comprehensive. The search terms used were “melasma” as a title word combined with the following additional terms individually within any field; “Asian,” “etiology,” “pathophysiology,” “prevention,” and “treatment.” A total of 135 articles were retrieved, duplicate results were excluded, and evaluated. 

Pathophysiology

Microscopic observation of epidermal keratinocytes obtained from patients with melasma demonstrates the abnormally large size, irregular borders, and altered chromatin structure [8]. These abnormalities may be caused by the effects of reactive oxygen species (ROS). An imbalance between ROS and antioxidant defense mechanisms can lead to the release of enzymes that cause oxidative stress leading to the development of melasma [9]. The abnormalities in chromatin structure and keratinocyte structure can be attributed to the buildup of ROS. Additionally, ROS has been implicated in the degradation of dermal connective tissue through increased expression of matrix-metalloproteinases which leads to skin aging and hyperpigmentation [9,10]. High levels of antioxidant enzymes such as superoxide dismutase, catalase, and glutathione peroxidase are used to assess oxidative damage. Malondialdehyde, the end-product of lipid peroxidase, can also be used to assess tissue damage due to ROS. Previous research tested the levels of these enzymes on patients with facial melasma and concluded that the levels of superoxide dismutase, catalase, glutathione peroxidase were significantly higher in melasma patients as compared to control patients (p<0.001), concluding that oxidative stress plays a role in the pathogenesis of melasma [11,12].

Chung et al. performed microarray analysis and polymerase chain reaction (PCR) on Korean women with melasma and compared those results to those obtained of women with healthy skin to identify the role of specific genes involved in its pathogenesis. Their analysis found 334 genes in the affected skin of these melasma patients that were significantly different than those in healthy women. The largest differences found in the gene expression profile of melasma patients included a nine-fold upregulation of guanine deaminase, a two-fold upregulation of homogentisate 1,2 dioxygenase, a 1.75-fold increase in tyrosinase-related protein 1, and a 1.27-fold increase in tyrosinase [13]. Contrastingly, genes involved in peroxisome proliferator-activated receptor (PPAR) signaling pathway such as adiponectin, C1Q, collagen domain-containing, adipocytes, and fatty acid-binding-protein 4 were down-regulated (>2 fold) in melasma patients [13]. 

With the downregulation of these proteins in the affected skin, it may be that melasma causes an impaired skin barrier function. Two factors that can be used to measure barrier function are transepidermal water loss (TEWL) and stratum corneum (SC) hydration. In a study consisting of 16 Korean patients with facial melasma, baseline SC hydration levels were significantly greater in lesioned skin, suggesting that melasma skin is thinner and more permeable [14]. This was further indicated by a decreased stratum corneum thickness in lesioned skin. TEWL measurements showed no significant differences between the melasma group and the control. However, greater TEWL was observed in melasma lesioned skin compared to healthy skin after the skin barrier was disturbed by tape stripping [14].

Additionally, epigenomic changes such as DNA methylation appear to have a role in melasma pathogenesis. DNA methylation is regulated by DNA methyltransferases (DNMT1, DNMT3a, DNMT3b). Increased expression of these methyltransferases may lead to greater expression of pro-inflammatory and pro-melanogenic genes. In an eight-week randomized double-blind controlled trial of 30 patients, it was found that there are significantly higher levels of DNMT1 on melasma skin as compared to healthy skin [15]. DNMT1 is implicated in the hypermethylation of DNA. Environmental factors such as ultraviolet (UV) radiation may also contribute to hyperpigmentation by activating pathways regulated by epigenetic modifications [15].

Since disease progression is also heavily influenced by pregnancy and by the fluctuations of female hormones, research has shown that estrogen upregulates PDZ domain protein kidney 1 (PDZK1) leading to increased tyrosinase activity which generates melanin and causes hyperpigmentation. Kim et al. discovered that the mRNA levels of PDZK1 were significantly higher in melasma skin as compared to healthy skin (p<0.015) and an anti-PDZ antibody showed greater reactivity in hyperpigmented skin [16]. Polycystic ovarian syndrome (PCOS) is a prevalent hormonal disorder characterized by high levels of estrogen causing oligomenorrhea. High levels of estrogen have also been shown to increase alpha-melanocyte-stimulating hormone which is correlated to melasma development, implying that women with oligomenorrhea may be at higher risk of developing melasma [17].

Potential risk factors

It has been noted that the primary risk factors for developing melasma are female hormone variations and chronic exposure to ultraviolet radiation [18]. Some other risk factors include genetics, chronic drug intake, and pollution [19].

Female sex hormone variations (increased or decreased levels of estrogen and/or progesterone) such as those induced by oral contraceptive pills or pregnancy are a major causative factor in the pathogenesis of melasma [20]. During pregnancy, in particular, immunologic, endocrine, and metabolic changes make pregnant women vulnerable to changes in the skin [21]. The mean age of onset is 38 years predominantly in women (85%) and of those, 59.2% reported onset during their second trimester of pregnancy and 34.6% reported onset after pregnancy [22]. This suggests that female hormone variations may be involved in the initial pathogenesis of melasma. In addition to pregnancy, melasma is also prevalent in women who are given progesterone-containing contraceptive pills making progesterone important risk factors in the disease [23]. This is interesting to note, as it is well known that estrogens may increase skin pigmentation, however, the prevalence of melasma in those who take progestin-only pills suggests there is an additional cause to the hyperpigmentation caused by melasma [23].

As melasma progresses, the effect of female hormone therapy on the progression of melasma is minimal, especially in patients with a familial history of melasma [19]. In a study of 45 patients with extra-facial melasma, there was no significant difference between the melasma and control groups in terms of the use of postmenopausal HRT and oral contraceptive pills [24]. This indicates that female hormone variation may be a primary risk factor in the pathogenesis of melasma, however additional research is required to determine the exact mechanism.

Additionally, UV is considered another one of the main triggering factors for the development of melasma [20]. This is postulated because UV-A, UV-B as well as visible light are all capable of stimulating melanogenesis [18]. In a clinical study that consisted of Asian Indians with a history of facial melasma, researchers were interested in the dependence of the severity of recurring melasma when the patients were exposed to certain risk factors. Of 10,001 patients who were studied, 85% were female and with a median age of 38 years and from 10 melasma treatment centers across India. The patients were surveyed and their duration of sun exposure per day was approximated. The estimated exposure time was then compared to the severity of the melasma [22]. The severity of melasma was determined using the Melasma Area and Severity Index (MASI) which is a universal tool that quantifies the severity based on three factors: area of involvement, darkness, and homogeneity [3]. A numeric score of 0-6, ranging from 0=no involvement to 6=90-100%, is calculated and assessed. It was concluded that there was a positive statistical difference between the duration of exposure to the melasma severity index (p<0.001) [22].

**Figure 1:**
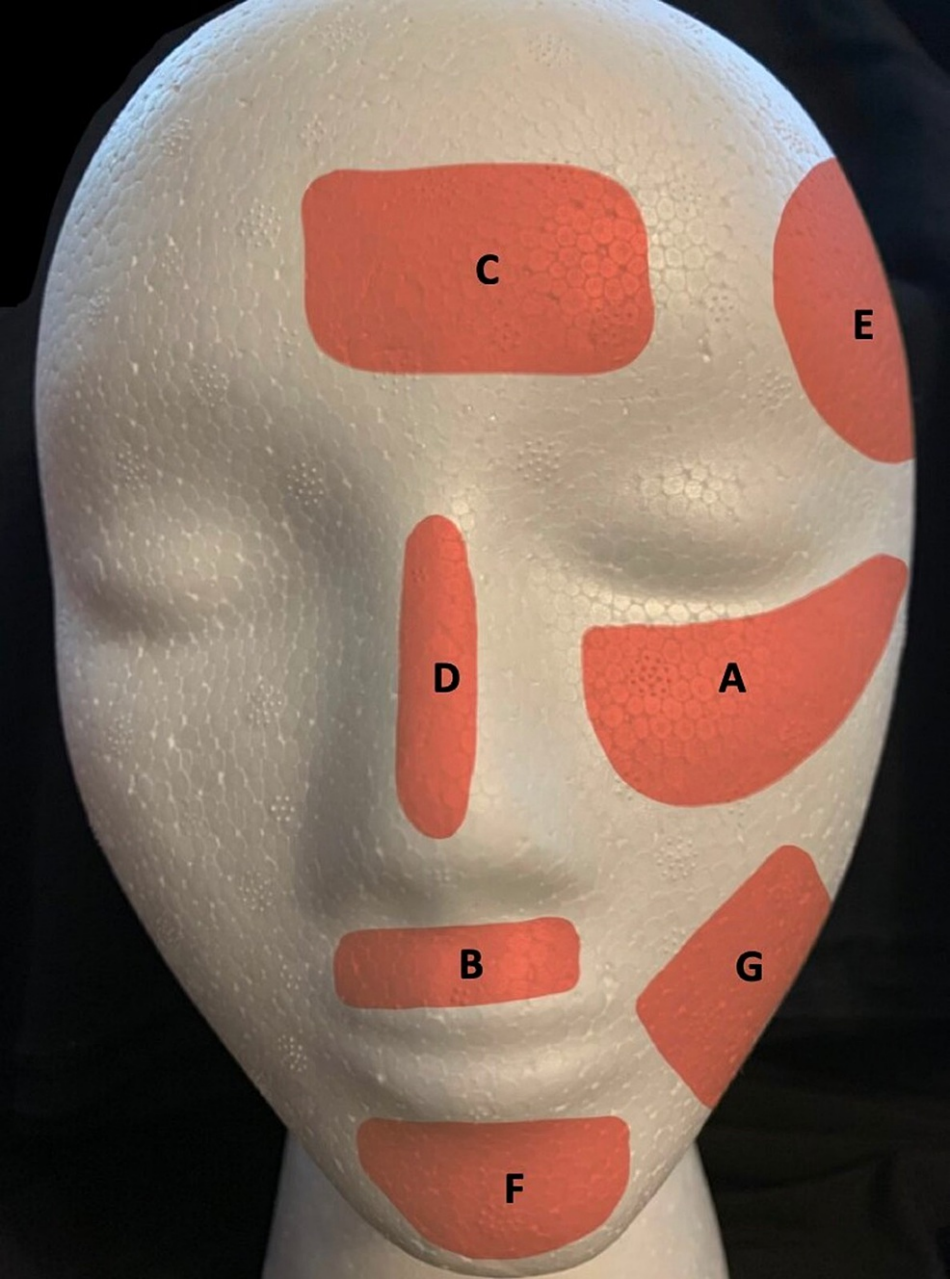
Facial areas most frequently affected by melasma. Shaded areas indicate locations of frequent melasma occurrence as determined in reference [19]  based on data from 209 patients. The location order (A through G) is from the most to least frequent location. The named areas are given along with prevalence percentages as follows: zygomatic (A-69%), supralabial (B-44%), frontal (C-41%), nasal (D-27%), temporal (E-20%), mental (F-19%), and mandibular (G-20%).

In addition to UV radiation, visible light is one of the causes of the initial onset and relapse of melasma by inducing skin hyperpigmentation in individuals with darker skin types [25]. The most common site of facial occurrence of melasma is the zygomatic region, followed by supralabial, frontal, nasal, temporal, mental, and mandibular areas as illustrated in Figure 1. These locations are facial areas that are more prone to UV and visible light exposure [20].

Limited evidence suggests family history influences melasma incidence. For example, a 2013 study noted that as many as 56.3% of 302 patients have a family history with melasma [26]. It was also noted that in a study of 207 individuals with melasma, 61% reported a family history of melasma, with sibling co-occurrence being the most common (35%) followed by parents (30%) [20]. These findings may suggest a potential genetic component to the onset of melasma. 

Additionally, there have been small studies that have reported cosmetic products and drugs that bind to melanin pigments may also cause melasma development. A case study documenting a 35-year-old male taking clomipramine, a tricyclic anti-depressant for obsessive-compulsive disorder noted a development of hyperpigmented spots on the malar eminences of the face a few days after starting the medication and his facial hyperpigmentation disappeared after cessation of the drug [27]. Another case report of drug-induced hyperpigmentation is the use of the tyrosine-kinase inhibitor imatinib, an approved drug for the treatment of cancers such as chronic myeloid leukemia, metastatic GI tumors, and malignant melanoma. Five patients on imatinib of 400mg daily reported melasma-like spots on their facial area as well as one individual with hyperpigmentation on their forearm [28]. This is interesting as there were no morphological differences noted between the melasma-like pigmentation induced by imatinib and melasma. With only a few cases available, the exact mechanism of imatinib-induced hyperpigmentation is still unclear. However, it is thought that c-kit, a constitutively active breakpoint cluster region-Abelson (BCR-ABL) protein targeted by imatinib, acts on microphthalmia transcription factor which is a protein critical for melanocyte development [29]. 

Another potential risk factor for the development of melasma is air pollution. Studies have shown a positive correlation between the prevalence of melasma and air pollution [9]. Pollutants in the form of airborne particulate matter and polycyclic aromatic hydrocarbons are said to enter the skin through nanoparticles to produce reactive oxygen species, which triggers mechanisms that favor accelerated aging, causing skin hyperpigmentation [9]. With Southeast Asia and the Indian subcontinent being one of the most polluted geographic areas, melasma and hyperpigmentation are noted to be the highest in those regions [9].

Prevention

As melasma is a condition that is heavily influenced by exogenous factors, prevention of disease is key. Behavioral modifications such as refraining from visits to the solarium during vulnerable states such as pregnancy, after the application of photosensitizing cosmetics, and/or following the intake of certain medications are important. In order to evaluate the importance of sun protection in pregnant women, Purim et. al performed interviews to discuss the correlation of photoprotection, melasma, and quality of life [30]. Only 34% of women interviewed had prenatal guidance on the importance of sun protection and of those individuals, a positive correlation between prenatal guidance and the use of daily sunscreen (p=0.088) and a lower prevalence of melasma was determined [30].

Prior research has also discussed serum zinc levels in dermatological disorders. In a total of 118 melasma patients, mean serum zinc levels were measured and compared with 118 healthy patients. Results showed that 45.8% of melasma patients had a serum zinc deficiency as compared to only 23.7% in control patients (p=0.001) [31]. Oral zinc supplements and zinc screening may be beneficial for the prevention of melasma in high-risk populations. Additionally, Vitamin D levels should be monitored as avoiding UV and sunlight exposure to prevent melasma may cause a decrease in serum Vitamin D. To ensure adequate Vitamin D levels despite avoiding UV sunlight, an oral Vitamin D supplement should be discussed at regular doctor visits [32]. In a study comparing melasma patients with other co-morbidities, there is a positive correlation between melasma and abnormal serum Vitamin D levels (p<0.050) [33]. 

The reoccurrence rate of melasma is high, especially when re-exposed to triggering factors such as oral contraceptive pills, pregnancy, and UV exposure. Researchers have tested the efficacy of a triple combination topical cream that contains fluocinolone acetonide, hydroquinone, and tretinoin. They found that 53% of patients remain relapse-free and that is also effective in postponing relapse in patients with severe outbreaks of melasma [34].

Treatments and complications 

Although melasma is a benign disorder that does not cause physical debilitation, it can negatively impact an individual’s psychological and emotional well-being if left untreated. The impact of melasma on quality of life is measured by the Melasma Quality of Life Scale (MelasQol) which is a questionnaire that consists of 10 questions regarding the effects of melasma on emotional well-being, social relationships, and daily activities of individuals [35]. For each question, patients rank on a scale of 1 (completely unbothered) to 7 (constantly bothered) and the total score is then calculated through statistical analysis [35]. In a study of 51 patients with melasma, 94.1% of patients felt bothered about their skin appearance with 56.9% of them feeling constantly bothered. 78.4% felt unattractive, 64.7% felt frustrated and embarrassed and 52.9% experienced depression from their skin appearance. Social relationships and restricted sense of freedom related to melasma were not significant (p>0.05) [35].

The use of lasers to reduce hyperpigmentation has been a popular treatment of choice for lightening melasma spots particularly in Asia [36]. Zhou et al. showed that a 694-nm fractional Q-switched ruby laser (QRSL) and sonophoresis on levorotatory Vitamin C (L-ascorbic acid) causes a significant improvement of melasma. After four sessions of treatment at two-week intervals, the mean MASI scores measuring severity decreased from 15.51 before treatment to 10.42 four weeks after final treatment (p<0.01) [37]. Also, combination treatment of intense pulsed light (IPL) and low-fluence 1064-nm Q-switched Nd:YAG laser on Korean patients have also proved efficacy [38]. Also, it has been shown that a 1064-nm Q-switched Nd:YAG laser has proven to be effective, well-tolerated, and decrease the melasma reoccurrence rate [38,39]. Interestingly, the decrease in MASI in the combination group was significantly larger than the intense pulsed light-only group (p<0.05) [38]. However, IPL alone has not been a popular option due to reports of pigment accentuation and reaggravation of melasma after repeated therapy, hence a novel fractionated IPL was developed due to its low-fluence properties and found a lower risk of rebound melasma and other adverse effects, proving to be a better alternative [40]. This fractionated IPL is not the mainstay of treatment for melasma, but it can be used as maintenance therapy. 

Additionally, a case study of combining the 2940-nm fractional Er:YAG and 1064-nm Q-switched Nd:YAG laser on two Chinese women with melasma showed rapid improvement of symptoms within 1 month of treatment and no complications during their 6 months follow up [41]. Q-switched Nd:YAG laser used along with a modified Jessner’s chemical peel has also proved to be highly effective in especially darker skin individuals with mixed melasma [42]. Advantages of this combination include lower-cost laser therapies and reduced potential common side effects after multiple laser sessions such as mild erythema and burning [42]. Another type of laser, the picosecond alexandrite laser at 750 nm has proven effective in the treatment of refractory melasma. Polnikorn et al. reported that 93% of patients who received the alexandrite laser every two weeks for three months were satisfied with the results with a melasma severity index drop from 6.22 to 1.48 after six months [43]. A few reported some side effects that include hypopigmentation, reoccurrence, and erythema [43]. 

Some topical treatments have proven to be effective in patients with melasma. Tranexamic acid works to inhibit plasminogen activation by blocking lysine-binding sites on plasminogen. It is commonly used to prevent hemorrhage and to treat menorrhagia but it also provides inhibitory effects on UV-induced plasminogen activator [44]. Therefore, tranexamic acid is a common treatment option for patients with melasma. Adverse effects with oral, systemic therapy of tranexamic acid include deep vein thrombosis and abdominal bloating are increased therefore topical preparations are preferred. Although tranexamic acid can be used alone, it is more commonly applied along in conjunction with lasers and intradermal injections among other modalities [44]. Xu et al. workers reported that the use of microneedles combined with topical tranexamic acid for 12 weeks resulted in a significantly lower melasma index and greater patient satisfaction in patients when compared to patients only treated with tranexamic acid [45].

Topical hydroquinone, a depigmenting agent, is also one of the common treatments for melasma [46]. However, chronic hydroquinone usage may have potential mutagenic risks and thus may need to be avoided [46]. Therefore, alternative depigmenting agents should be considered. A case report suggests that methimazole may reduce melasma spots in patients due to its depigmenting properties. The advantages of methimazole are that it is odorless, well-tolerated by individuals, and not associated with any cytotoxic or mutagenic effects [46]. Truchuelo et al. discussed a new formula called Neoretín® which encompasses retinoids including retinol glycospheres and hydroxypinacolone retinoate and depigmenting agents has demonstrated success in treating melasma-like hyperpigmentation. Patients’ hyperpigmented spots were reduced in 89% of the treated area while only 56% of the area showed some improvement in the control group who received a vehicle formulation (p<0.001) [47]. Neoretín® was also well-tolerated except for a few patients who reported intense pruritis, burning sensation, and erythema [47].

Based on a known correlation of melasma and oligomenorrheic states including pregnancy, it was discovered that menstruation-induced systemic therapies are effective in treating the disease, and maybe more effective than topical treatments in the long run [17]. A case report consisting of four women showed that their melasma spontaneously improved upon switching from estrogen and progesterone combined oral contraceptive pills (COCP) to a levonorgestrel-releasing intrauterine device (IUD) [48]. The clear reason for the improvement is unknown but researchers suggest it is partially due to the extensive amount of estrogen produced by COCP while the IUD releases only a small amount of levonorgestrel [48]. Topical hormone-based therapies for melasma include anti-estrogenic and aromatase inhibitors which have been shown success in treating the condition [49]. Examples of anti-estrogenic medications include Tamoxifen and raloxifene which have been approved for breast cancer prevention in postmenopausal women. An example of aromatase inhibitors is exemestane, a medication that halts the conversion of testosterone to estrogen, in treating breast cancer, leiomyoma, and short stature in women. These may be considered as alternative therapies for melasma. However, there are significant adverse effects associated with these conditions such as endometrial carcinoma, and should be used conservatively [49]. 

Discussion 

Risk factors for the development of melasma include hormonal variations, ultraviolet radiation, and visible light, chronic drug intake, and air pollution [18-20]. Since melasma pathogenesis remains unclear, it is important to avoid exposure to these risk factors such as sun avoidance during pregnancy. Zinc deficiency is associated with the development of melasma, therefore monitoring zinc levels at annual follow-up visits may be useful in the prevention of melasma [31]. Although there is much research dedicated to finding treatments for melasma, reoccurrence is common and in order to maximize the quality of life, prevention is the most efficient method to consider for high-risk individuals. 

The present review uncovered insufficient evidence of the effects of drugs such as clomipramine and imipramine on its correlation with the development of melasma as there are only two cases reported [27,28]. Additional research is warranted on the effects of clomipramine in particular on inducing melasma-like rashes in female patients. This will further help characterize an association with pharmacotherapy in the pathogenesis of melasma.

Furthermore, there are limited studies on TEWL and SC hydration measurements in melasma patients. More research on these can help determine the degree of impairment in skin barrier function associated with melasma. Additionally, research on gene methylation is sparse and further work may be warranted, including a focus on specific environmental, and lifestyle changes that can lead to these methylations.

Further research is also needed to determine the significance of prenatal guidance on the use of sunscreen as it may prompt more vulnerable individuals to protect from and avoid UV light. Studies should be conducted to determine if these protective measures cause a significant decrease in Vitamin D levels requiring supplementation, particularly within the prenatal period.

## Conclusions

The present review has confirmed that although melasma does not usually cause physical debilitation, many patients report experiencing depression, and decreased quality of life making melasma an important condition to characterize and educate both healthcare providers and patients. Since melasma is a hyper-pigmentary condition that commonly appears on the skin of Asian women and to some extent in individuals with darker phenotypes, these groups are especially at risk and should be targeted for enhanced educational effort. Although the exact mechanism of the pathogenesis of melasma is unknown, this review has found compelling evidence that high levels of antioxidant enzymes in melasma tissue with associated oxidative stress may play a role. Finally, based on the evidence that melasma-related skin changes may impact skin barrier functions a natural follow-up clinical study would be to systematically investigate the relationship of melasma severity to alterations in TEWL as a direct measure of such compromised barrier function. Such studies are presently being developed.
